# Metabolomic characterization and temporal dynamics of *Burkholderia cepacia* for polyhydroxyalkanoates production from oleic acid

**DOI:** 10.1007/s11306-026-02473-x

**Published:** 2026-06-16

**Authors:** Alfonso E. Alarcón, Nubia C. Moreno, Daniel Pardo-Rodriguez, Mónica P. Cala, Carlos A. M. Riascos

**Affiliations:** 1 Chemical and Environmental Engineering Department, Universidad Nacional de Colombia, Bogotá, Colombia; 2 Institute of Biotechnology, Universidad Nacional de Colombia, Bogotá, Colombia; 3 Metabolomics Core Facility - MetCore, Vice-Presidency for Research, Universidad de los Andes, Bogotá, Colombia

**Keywords:** Polyhydroxyalkanoates, *Burkholderia cepacia*, Untargeted metabolomics analysis, Dynamic analysis

## Abstract

**Background:**

Polyhydroxyalkanoates (PHA) have become biodegradable alternatives for replacing chemical polymers. However, high production costs remain the primary obstacle to the commercial application of this bioprocess, prompting the use of metabolic engineering approaches to optimize PHA quality and increase productivity.

**Objectives:**

This study aimed to identify the endometabolome at different stages during a dynamic fermentation of *Burkholderia cepacia*using oleic acid as the carbon source.

**Methodology:**

Untargeted metabolomic analysis was conducted using gas chromatography-mass spectrometry (GC-MS) and liquid chromatography-mass spectrometry (LC-MS) techniques. Univariate (UVA) and multivariate (MVA) statistical analyses were performed to determine significant differences between metabolomic profiles.

**Results:**

A total of 24 significant metabolites were identified through GC-MS analysis and 223 through LC-MS, highlighting organic compounds and lipids associated with pathways such as ß-oxidation, the tricarboxylic acid cycle, and the pentose phosphate pathway. Acetyl-CoA emerged as a critical common intermediate in both central biosynthetic and PHA-producing pathways.

**Conclusion:**

Acetyl-CoA plays a fundamental role in the complex regulatory system activated by nutrient fluctuations in the culture medium, suggesting that the microorganism undergoes metabolic reorganization to optimize carbon source utilization for cellular maintenance.

**Supplementary Information:**

The online version contains supplementary material available at https://doi.org/10.1007/s11306-026-02473-x.

## Introduction

The pollution generated by petroleum-derived polymers and their recycling difficulties has driven the need for new and greener materials (Emadian et al., 2017; Mohapatra et al., 2017; Peptu & Kowalczuk, 2018). Polyhydroxyalkanoates (PHA) have become biodegradable candidates for the replacement of chemical polymers due to their similarity in properties with conventional plastic materials (tensile strength and flexibility) and their complete biodegradability (Kumar et al., 2020; Nielsen & Keasling, 2016; Suriyamongkol et al., 2007; Urtuvia et al., 2014). Microorganisms make PHA inside their cells and store them as an energy source (Możejko-Ciesielska & Kiewisz, 2016; Sagong et al., 2018). Among the bacterial species capable of accumulating large quantities of PHA and its copolymers are *Cupriavidus necator*, *Pseudomonas sp.*, and *Burkholderia sp*. (Anjum et al., 2016; Chee et al., 2010; Urtuvia et al., 2014; Zhu et al., 2010).

One of the main obstacles to the commercial application of PHA production bioprocesses is the high production cost (Bano et al., 2024; Mitra et al., 2020). Profitability is directly related to the microbial rate of growth and production, the substrate cost, and the substrate conversion efficiency (Albuquerque & Malafaia, 2018). However, Akiyama et al. (2003) determined that using vegetable oils as a carbon source generates higher PHA yields (0.6–0.8 g of PHA per g of oil) than using simple sugars. In addition, some tested strategies for improving process profitability are: prospecting for new strains (Cerrone et al., 2023; Kucera et al., 2018), genetic improvement to obtain super-producing strains (Hernández Jirón, 2023; Santolin et al., 2024), and formulation of metabolically structured models to support process optimization (Torres Ospina, 2019).

Previous studies, by our research group, on PHA production with *B. cepacia* (Ardila Arévalo & Viloria García, 2017; Becerra Jiménez, 2013; Méndez, 2016; Viloria et al., 2017) showed that achieving higher biomass concentrations is necessary to improve the profitability of the process. Additionally, in the work of Torres-Ospina & Riascos (2020), through fluxomics at different times of a batch fermentation with oleic acid, using a simplified metabolic network it was possible to observe that during the growth phase 61% of the carbon is used for the generation of biomass and energy, while 39% for PHA generation. By contrast, during the production phase, carbon distribution changes to 43% and 57% respectively, reaching a PHA/biomass ratio of 77% by weight. From these findings, it was concluded that the metabolic “machinery” undergoes important changes between the two phases and that these changes are not instantaneous, as it implies an adjustment in metabolic functioning. Due to the metabolic implications of the phase shift, to extend the growth phase requires the development of metabolic regulation strategies.

In another effort for developing commercially competitive processes, synthetic biology and metabolic engineering techniques were used to improve PHA qualities and increase bioprocess yield (Bano et al., 2024). Metabolic engineering is based on the concept of a metabolic network as a sequence of reactions catalyzed by specific enzymes that convert substrates into cellular products (Sharma et al., 2019; Stephanopoulos & Sinskey, 1993). The metabolic network plays a crucial role in cellular physiology (Litsios et al., 2018; Min Lee et al., 2008), as it involves different regulatory mechanisms that control cellular activities through timely and appropriate physiological modifications in response to environmental changes (Gerosa & Sauer, 2011; Litsios et al., 2018; Shen et al., 2016; Stephanopoulos, 1999). Therefore, studying intracellular metabolic processes offers alternatives to efficiently manipulate the metabolic pathways to improve bioprocess.

Furthermore, intracellular metabolite concentrations are critical to understand metabolic dynamics, providing insight into the biochemical mechanisms operating within the system. One tool for identifying and quantifying metabolites in a biological system is metabolomics (Luo et al., 2007; Xiao et al., 2012), this technique allows to infer metabolic changes in a microorganism from determining metabolite concentrations at various times during cell culture (Buchholz et al., 2002; Chen et al., 2025; Čuperlović-Culf et al., 2010). Among the most used methods for identification and quantification of metabolites in microbial metabolomics are mass spectrometry (MS) coupled with separation techniques, such as gas chromatography-mass spectrometry (GC-MS) and liquid chromatography-mass spectrometry (LC-MS), due to the sensitivity and separation efficiency (Ye et al., 2022).

The metabolic changes, inferred from the metabolomic analysis, contribute to a better understanding of the metabolic dynamics. In a recent review, Tanaka et al. (2024) confirms metabolomics as a fundamental tool into the DBTL cycle. The Design-Built-Test-Learn (DBTL) cycle is a workflow strategy for creating high-producing microbial strains with the aim of achieving commercial scale production. Additional to strain improvement, the optimization of cultivation conditions can also benefit from the detailed knowledge that metabolomics generates about the behavior of metabolism, as supported in the review of Tanaka et al. (2024).

Given the problems posed by the high costs associated with PHAs production process and its low competitiveness, metabolomics becomes an alternative to increase the knowledge of the metabolic system and its regulatory mechanisms and to propose strategies that make the bioprocess more commercially competitive. In the present work an untargeted metabolomic analysis was conducted at different time points during a batch fermentation of a *Burkholderia cepacia* strain with oleic acid as the carbon source. The aim was to characterize the metabolic alterations occurring throughout the cultivation process and to elucidate their association with the main metabolic pathways used by the microorganism at distinct growth stages. This type of analysis provides valuable information about microbial metabolism, helping to identify bottleneck into the pathways, which in future works will lead the improvement in PHAs production, as part of the DBTL cycle.

## Materials and methods

### Chemicals

For sample preparation and chromatographic analysis, high-grade methanol, n-Heptane and acetonitrile for liquid chromatography coupled to mass spectrometry (LC-MS) LiChrosolv^®^ were purchased from Merck Millipore (Massachusetts, USA), as were the formic acid for LC-MS LiChropur™. N, O-Bis(trimethylsilyl)trifluoroacetamide with 1% trimethylchlorosilane LiChropur™, Methoxyamine hydrochloride with a purity of 98%, Methyl heptadecanoate-d33 in heptane internal standard, Fatty Acid Methyl Ester (FAME) organic reference standard Supelco^®^, Alkanes from C8 to C20 analytical standard was purchased from Sigma-Aldrich (St. Louis, United States).

### Fermentation

#### Strain and culture conditions

For this study, a modified strain of *B. cepacia* was used. *B. cepacia* B27 is a strain obtained by spontaneous mutation at the Institute of Biotechnology, Universidad Nacional de Colombia (Moreno et al., 2007), as part of an extended effort to develop a commercial process for PHA production. This strain accumulates PHAs when grown on vegetable oils as carbon source; however, to simplify omics analyses (fluxomic in Torres and Riascos, 2020, and metabolomics in the present work) oleic acid was used as the sole carbon source. Cells were grown in a defined fermentation medium containing Na₂HPO₄ 3.39 g/L, (NH₄)₂SO₄ 2.8 g/L, MgSO₄·7 H₂O 0.3 g/L, KH₂PO₄ 2.65 g/L, micronutrient solution 2 mL/L, and oleic acid 20 g/L.

Strain activation was performed according to the methodology proposed by Torres-Ospina (2019). Briefly, 1 mL of the frozen culture stock was inoculated into 10 mL of LB medium and incubated at 30 °C for 24. The resulting culture was then transferred to 100 mL of fermentation medium and incubated at 30 °C for 12 h. Finally, this pre-culture was inoculated into a Biostat^®^ bioreactor with a working volume of 1 L and operated at 30 °C, 9 g agitation, pH 6.5 and 2 vvm of aeration for 24 h. Dissolved oxygen was monitored using an Oxymax COS22D dissolved oxygen sensor.

####  Sampling

Six biological replicates were collected from independent fermentations, each consisting of 50 mL of fermentation broth. Samples were collected at 6, 12, 16, and 22 h post-inoculation.

Immediately after sampling, cells were rapidly quenched by adding a 20% v/v methanol–water solution at 4 °C in a 3:1 ratio relative to the sampling volume, followed by centrifugation at 4 °C and 7000 g for 7 min. The resulting cell pellets were then washed with 50 mL of phosphate-buffered saline (PBS) at 4 °C, homogenized by vortexing for 30 s, and centrifuged again; the supernatant was discarded, and the washing step was repeated once. The samples were thus homogenized by dilution, and the final cell pellets were stored at − 80 °C until analysis (Marques & Justino, 2023; Patejko et al., 2017; Van Gulik et al., 2013).

### Sample analysis

#### Sample preparation

Samples were extracted with acetonitrile-methanol-water solution (40:40:20), homogenized by vortex for 10 min, centrifuged at 4 °C and 24,000 g for 10 min, the supernatant was collected and passed through Agilent Technologies PTFE 13 mm, 0.2 μm filters, and finally aliquots of 50 µL per sample were placed in glass vials and stored at −80 °C until analysis by Liquid Chromatography Coupled to Mass Spectrometry with Time-Of-Flight *(*LC-QTOF-MS) and Gas Chromatography Coupled to Mass Spectrometry with Time-Of-Flight (GC-QTOF-MS) (Jaiyesimi et al., 2021; Marques & Justino, 2023).

For GC-QTOF-MS, 30 µL of supernatant was collected and evaporated to dryness for 1 h at 35 °C using a SpeedVac concentrator system. Then 10 µL of O-methoxyamine in pyridine (15 mg/mL) was added, vortexed for 5 min and incubated in darkness at room temperature for 16 h (Rey-Stolle et al., 2022). Silylation was performed by adding 10 µL of N, O-bis(trimethylsilyl)fluoroacetamide with 1% trimethylchlorosilane, vortex for 5 min, and incubated at 70 °C for 1 h. Finally, the samples were enabled to cool at room temperature for 30 min, 60 µL of methyl heptadecanoate D33 in heptane was added as internal standard (2 mg/L) and vortexed for 5 min (Rey-Stolle et al., 2022).

#### Reversed‑phase liquid chromatography–quadrupole time‑of‑flight mass spectrometry (RP‑LC–QTOF–MS) analysis

Untargeted metabolomic analysis was performed using an Agilent 1260 Infinity LC system coupled to an Agilent 6545 Q-TOF mass spectrometer (Agilent Technologies, Palo Alto, CA, USA). A 10 µL aliquot of the extracted sample was injected onto an InfinityLab Poroshell 120 EC-C_18_ column (2.1 × 150 mm, 2.7 μm; Agilent), thermostatted at 30 °C, using a binary mobile phase consisting of 0.1% (v/v) formic acid in Milli-Q water (phase A) and 0.1% (v/v) formic acid in acetonitrile (phase B) at a constant flow rate of 0.4 mL min⁻¹. The chromatographic method was adapted from (León-Inga et al., 2024) with the following modifications: the elution gradient started at 2% B, was increased linearly to 30% B at 10 min, then further increased to 98% B at 20 min and held for 2 min, after which initial conditions were restored with a re-equilibration time of 5 min. Mass spectrometric detection was performed in both positive and negative electrospray ionization (ESI) modes over an m/z range of 50–1100. Continuous mass axis calibration was achieved using two reference masses in each polarity: for positive mode, m/z 121.0509 (purine, [C_5_H_4_N_4_+H]⁺) and 922.0098 (HP-0921, [C_18_H_18_O_6_N_3_P_3_F_24_+H]⁺), and for negative mode, m/z 112.9856 (TFA(NH_4_), [C_2_O_2_F_3_(NH_4_)–H]⁻) and 1033.9881 (HP-0921, [C_18_H_18_O_6_N_3_P_3_F_24_+CF_3_COOH–H]⁻). Nitrogen was used as nebulizing and collision gas, with a nebulizer pressure of 50 psi, drying gas temperature of 325 °C and flow rate of 8 L min⁻¹, and sheath gas temperature of 350 °C and flow rate of 11 L min⁻¹; the capillary voltage was set to 3000 V and the fragmentor voltage to 175 V. Data were acquired in centroid mode at a scan rate of 1.0 spectra s⁻¹.

#### Gas chromatography–quadrupole time-of-flight mass spectrometry (GC–QTOF–MS) analysis

A 7890B gas chromatograph coupled to a GC/Q-TOF 7250 time-of-flight mass spectrometer (Agilent Technologies, Palo Alto, CA, USA) equipped with a split/splitless injection port (250 °C, split ratio 30) and an Agilent 7693 A autosampler was used for data acquisition, with the electron ionization source operated at 70 eV. A 1 µL aliquot of the derivatized sample was injected onto an Agilent J&W HP-5MS column (30 m × 0.25 mm × 0.25 μm), using helium as the carrier gas at a constant flow rate of 0.7 mL min⁻¹. The oven temperature was initially held at 60 °C for 1 min and then increased at 10 °C min⁻¹ to 325 °C, while the transfer line, ion source, and quadrupole temperatures were maintained at 280 °C, 230 °C, and 150 °C, respectively, and mass spectra were acquired from m/z 50 to 600 at 5 spectra s⁻¹ (Rey-Stolle et al., 2022). In the GC-QTOF-MS workflow, 50 µL of methyl stearate in heptane (C18:0, 10 mg L⁻¹) were added to each sample as an internal standard prior to analysis, followed by vortex mixing for 10 min at 5500 g, to monitor instrumental stability and to assess the reproducibility of injection, derivatization, and chromatographic response.

#### Quality control samples

Quality control (QC) samples were prepared by mixing equal volumes of extracted samples. To determine the reproducibility and stability of the analytical platforms used, several QC runs were performed before all samples were analyzed until equilibrium of the system was achieved and every fourth sample randomized (Dudzik et al., 2018; Kirwan et al., 2022; Mosley et al., 2024).

### Data processing

All raw data were processed as previously reported by Cala et al. (2019). Briefly, LC‑QTOF‑MS data were processed with Agilent MassHunter Profinder B.10.0 for deconvolution, alignment, and integration. GC-MS data processing consisted of a deconvolution step with Agilent MassHunter Unknowns Analysis B.10.00 and metabolite identification using Fiehn libraries version 2015 and NIST17. Agilent Mass Profiler Professional B.12.1 was used for retention time alignment, and peak integration for each metabolite was subsequently performed in Agilent MassHunter Quantitative B.10.00 following the manufacturer’s guidelines. Both LC-MS and GC-MS data were manually inspected to remove noise. The data were then filtered for presence and reproducibility, retaining metabolites detected in 100% of the biological replicates within each group and with a coefficient of variation in QC samples below 20%. In addition, prior to statistical analysis, total area normalization was applied to each sample individually. In this approach, the intensity of each feature was scaled relative to the total signal detected in its own sample, with the aim of correcting systematic analytical variation, such as differences in overall signal intensity, ionization efficiency, or injection volume. This procedure improves comparability across samples by reducing technical variability without relying on an external reference.

### Statistical analysis

To determine statistically significant differences between metabolomic profiles, univariate (UVA) and multivariate (MVA) statistical analyses were performed (Cambiaghi et al., 2017; Liland, 2011; Pakkir Shah et al., 2024; Vinaixa et al., 2012). Group comparisons were conducted using one‑way ANOVA, and p‑values were corrected for multiple testing using the Benjamini–Hochberg false discovery rate (FDR) procedure; both raw and FDR‑adjusted p‑values are reported. Principal Component Analysis (PCA) was applied as a preprocessing step that reduces data dimensionality, in that way the first and second component can be used to evaluate the quality of the acquired data, verifying that the quality control samples were correctly grouped in these models to ensure the stability of the analytical system (Broadhurst et al., 2018; Herrera-Rocha et al., 2021; Uarrota et al., 2014; Worley & Powers, 2016). Subsequently, Partial Least Squares Discriminant Analysis (PLS-DA) models were built to maximize and inspect the differences between the study groups and select the metabolites responsible for separating the groups; model performance was assessed through permutation and cross-validation tests (Westad & Marini, 2015; Westerhuis et al., 2010; Worley & Powers, 2013). Before statistical analysis, data auto-scaling was used (Pang et al., 2021). Significant variables were selected for all platform data only based on the following two criteria: (1) MVA criteria (significant Variance in Projection (VIP) > 1, and p values < 0.05 from the UVA test, and (2) Fold Change (FC) > 1 for increased metabolites and FC < 1 for decreased metabolites (Farrés et al., 2015; Vinaixa et al., 2012). All analyses were conducted using the MetaboAnalyst 5.0 server (Pang et al., 2021).

### Metabolite identification

The initial monoisotopic mass search was performed using the CEU Mass Mediator tool (Gil de la Fuente et al., 2018) and compared with the following online databases: Human Metabolome Database (http://hmdb.ca), MassBank (https://massbank.eu/MassBank/), Lipid MAPS (http://lipidmaps.org), GNPS, BioCyc (Karp et al., 2018), METLIN (Montenegro-Burke et al., 2020) and KEGG (Aoki-Kinoshita, 2006). MS/MS analysis was performed by automated annotation using MSDIAL (Lai et al., 2018), SIRIUS (Dührkop et al., 2019), MZmine and Lipid Annotator software, followed by manual inspection using Qualitative Analysis of MassHunter Acquisition Data version 10.0 software for spectral visualization. The identification level of the altered metabolites is described following the guidelines outlined by Blaženović et al. (2018).

### Metabolite pathway mapping

Metabolic pathway analysis was performed using the MetaboAnalyst 5.0 tool (http://www.metaboanalyst.ca/), which integrates two pathway analysis approaches, enrichment analysis and pathway topology analysis. A list of KEGG IDs of the metabolites identified using the *Burkholderia mallei* ATCC 23,344 (KEGG) library was loaded and processed (Pang et al., 2021; Xia & Wishart, 2011). KEGG pathways from *Burkholderia mallei* were initially used as a reference because this organism is annotated in KEGG as a reference genome with a well‑curated and comprehensive set of metabolic pathways within the *Burkholderia* genus.

### PHA quantification

Polyhydroxyalkanoate quantification was performed according to the protocol described by Torres-Ospina (2019). A 15 mL aliquot of fermentation broth was collected and centrifuged at 7000 g for 10 min. The pellet was washed twice with distilled water and then resuspended in distilled water containing 20% (w/v) sodium dodecyl sulfate (SDS) at a ratio of 0.55 mL per gB_X_. The samples were digested at 90 °C for 1 h. Subsequently, the samples were centrifuged again, washed, and dried at 80 °C for 24 h.

The volume of 20% SDS solution to be added was calculated using the following equation:

$$X_{{SDS}} \, = \,m_{{Bx}} (mg)\,*\,(0.55mL\,solution/100mg)$$

where X_SDS_ is the volume (mL) of the 20% SDS solution to be added, and m_Bx_ is the biomass mass in the sample (mg).

## Results and discussion

### Definition of sampling times

**Fig. 1:**
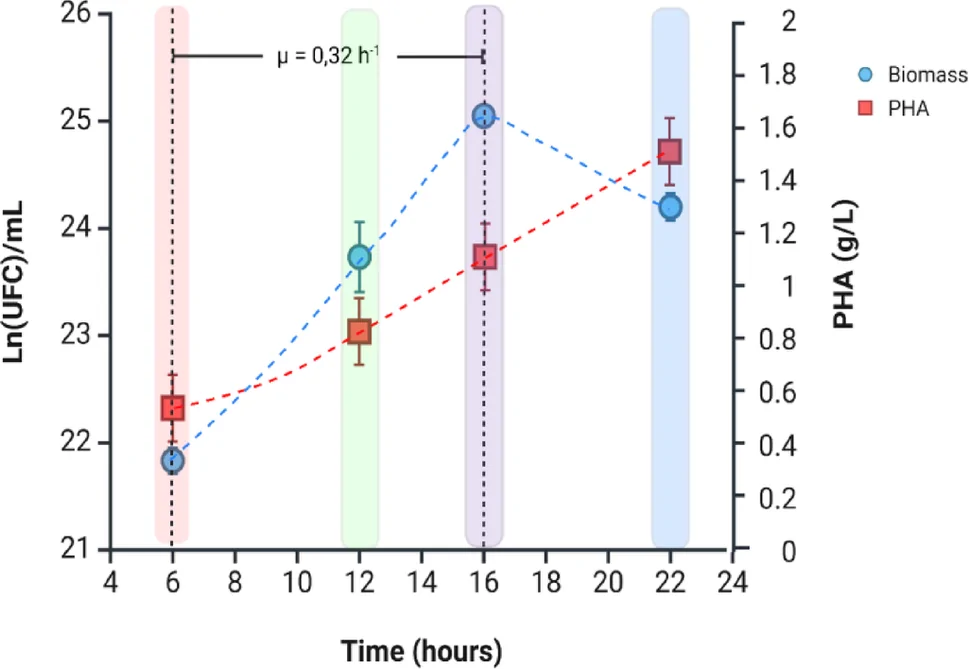
Growth phases and sampling times for metabolomics in B. cepacia using oleic acid as a carbon source in batch culture. Six replicates were taken for each sampling time and the means were graphed. The bars represent the standard deviation. The dotted lines delimit the exponential growth phase

The metabolic switch proposed by Torres Ospina and Riascos (2020) can be monitored by defining the sampling time points. Through a fluxomic study, in this previous work it was observed that the metabolic pathways adjust to direct carbon toward biomass production during the exponential phase, reaching 87% of total. However, this flux decreases to 14% during the stationary phase, indicating that the metabolic machinery of *B. cepacia* was reorganized in accordance with the nutrient concentrations in the culture medium during the exponential and stationary growth phases. In the present work, this metabolic switch is analyzed from the metabolomic point of view. The growth curve of *B. cepacia* was established using oleic acid as a carbon source, and the growth rate was measured for each of these moments. This allows to define the metabolic phases of the bioprocess. As shown in Fig. 1, the growth rate between 6 and 16 h of culture is constant (µ = 0.32 h^− 1^), establishing that the bioprocess is in exponential growth phase. At 22 h of culture, a decrease in the specific growth rate was observed (µ = 0.16 h^− 1^), indicating that a change from exponential to stationary growth phase is happening. Based on this observation, the establishment of these culture phases allowed defining two sampling times within the exponential phase (6 and 12 h) another in the transient (16 h) and the last one in stationary phase (22 h). These analysis times will allow to observe the behavior of the metabolite concentrations and correlate them with the activity of the main metabolic pathways during the culture. PHA production was also observed in all the assessed times, where an almost constant accumulation is observed between 6 and 22 h of culture. This confirms that, in *B. cepacia*, PHA production begins at the onset of the exponential phase.

### Quality control analysis

Unsupervised PCA models were applied to evaluate the behavior of the QC samples (Broadhurst et al., 2018). All analytical platforms showed QC clustering, ensuring the consistency of the acquired data and confirming that group separation was related to biological rather than analytical variations (see supplementary material S1. Statistical analysis).

**Fig. 2:**
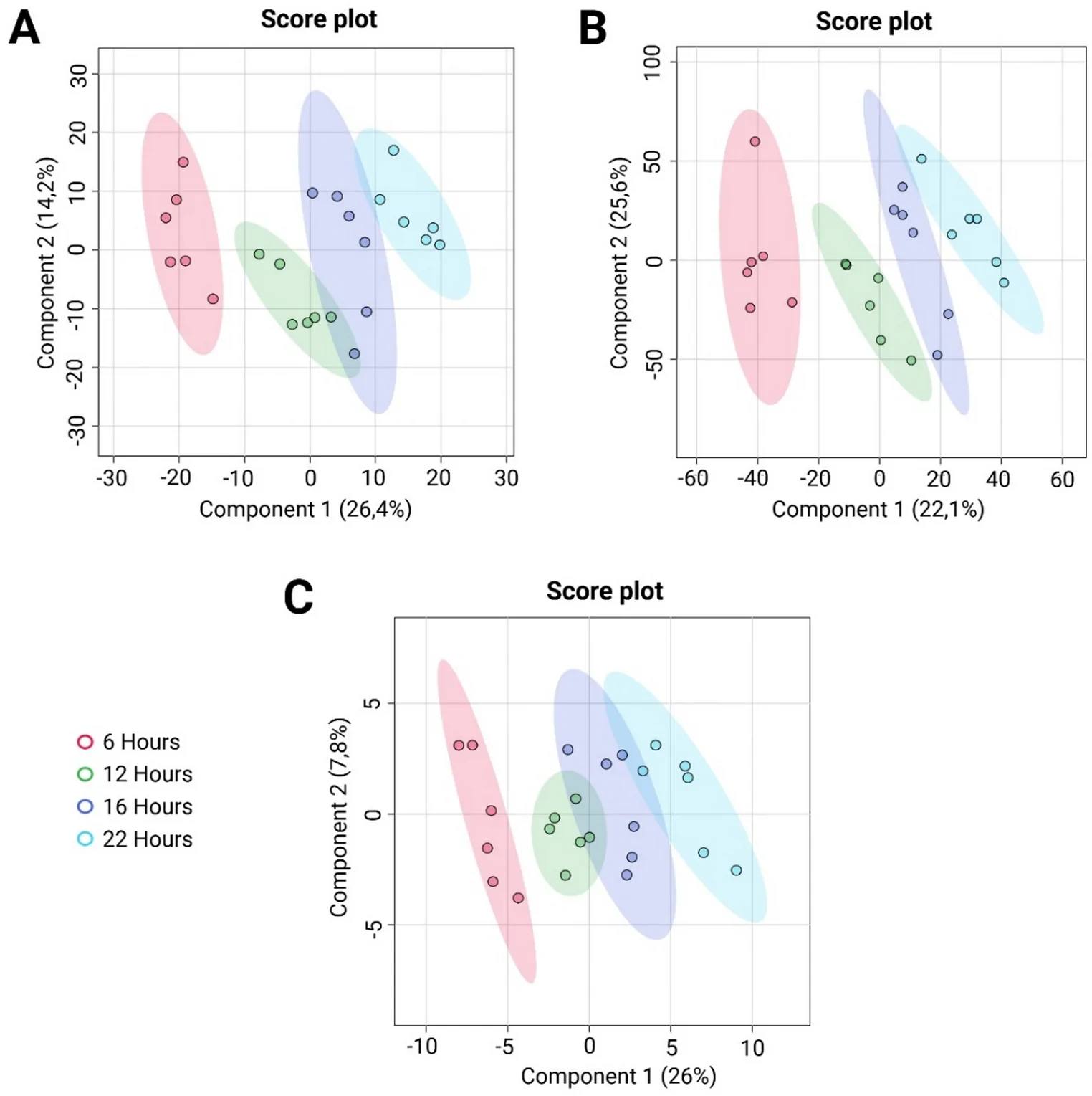
Multivariate PLS-DA analysis for the metabolome comparison between different fermentation times. **A** LC-QTOF-MS(+): R^2^= 0.96. Q^2^ = 0.90. **B** LC-QTOF-MS(-): R^2^ = 0.99. Q^2^ = 0.91. **C** GC-QTOF-MS: R^2^ = 0.95. Q^2^ = 0.89. The four ovals of different colors group the samples from different fermentation times (red 6 h, green 12 h, purple 16 h, and cyan 22 h), and the six circles inside each oval correspond to the replicates of each time point

The differences among experimental groups throughout the fermentation process, explored and maximized by partial least squares discriminant analysis (PLS-DA) models, are presented in Fig. 2. The models generated from the three analytical platforms revealed a clear separation of the metabolic profiles corresponding to the different times of the fermentation (6, 12, 16, and 22 h). This separation is consistently observed across all three analytical platforms, indicating significant metabolic differences at each stage of bacterial development, and agreement among the analytical platforms.

In addition to the significant separation of the groups, the sequential arrangement of the clusters along the plot of principal components reflects a temporal progression in the metabolic fingerprint, consistent with the expected physiological changes during *B. cepacia* batch fermentation. This progressive metabolic shift supports the notion of a dynamic metabolic reprogramming as fermentation advances. The robustness of the metabolomic results is supported by high values for the coefficient of determination (R² from 0.95 to 0.99) that quantifies the model´s capacity to correlate the observations: variance in metabolites abundance as function of the time in the fermentation, and for the cross-validated coefficient of determination (Q² from 0.89 to 0.91) that quantifies the model´s predictive capacity, these validation metrics confirm the statistical reliability of the analysis. The changes in metabolite abundance can be contrasted with the nutrient dynamics observed during the fermentation process, which progressively decrease over time and can induce changes in cellular metabolism, reflected on changes in metabolite concentrations during fermentation. Similar behavior has been previously described by other authors (Behera et al., 2022; Koller, 2020; Torres-Ospina & Riascos, 2020).

### Metabolite profiling at different fermentation times using multiple platforms

**Fig. 3:**
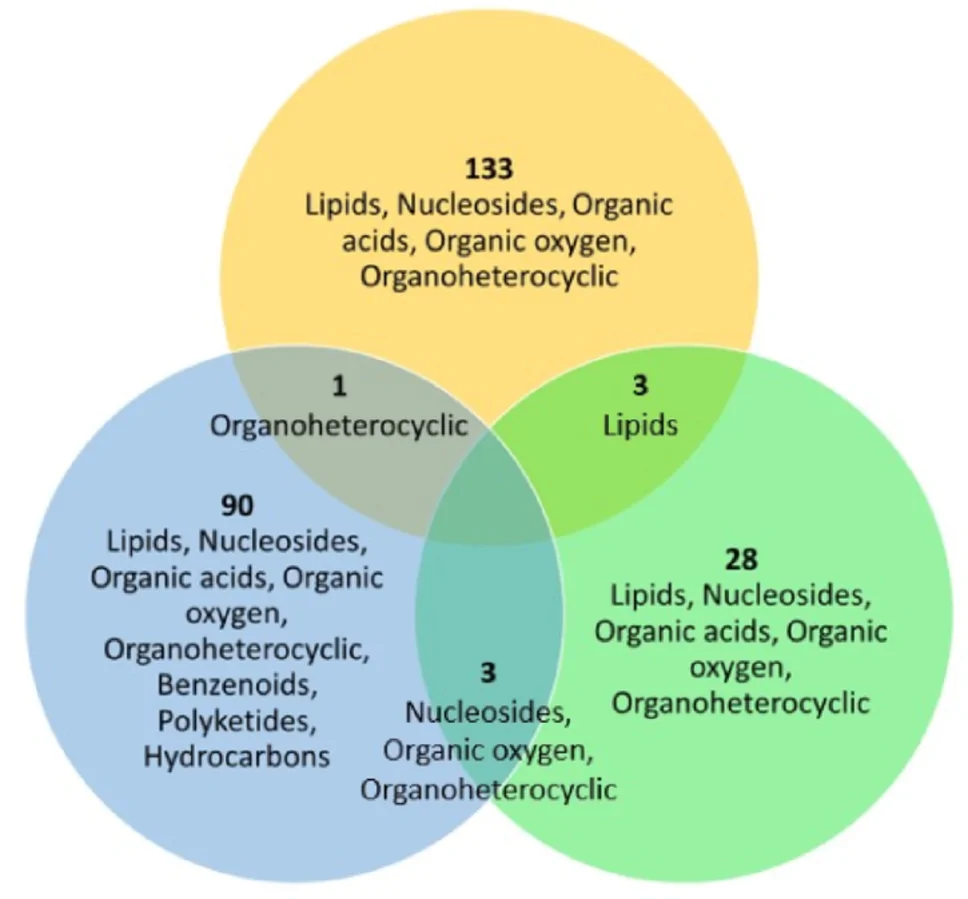
Metabolites identified by different platforms. Venn diagram showing the number of metabolites identified by each analytical platform: LC-QTOF-MS(+) (blue), LC-QTOF-MS(–) (yellow), and GC-QTOF-MS (green)

Statistically significant features were determined by a combination of MVA (VIP > 1 with JK) and UVA (percentage change > 20% and *p* < 0.05) criteria (see supplementary material S2. Table Metabolomics fermentation). Figure 3 presents a Venn diagram illustrating the number of altered metabolites identified by each analytical platform, classified by chemical family. A total of 133 metabolites were uniquely detected in the LC-QTOF-MS(-) platform (yellow), mainly belonging to lipid, nucleoside, organic acid, organic oxygen, and organoheterocyclic compound classes. The LC-QTOF-MS(+) platform (blue) identified 90 unique metabolites, showing greater chemical diversity including benzenoids, polyketides, and hydrocarbons, in addition to the previously mentioned classes. GC-QTOF-MS (green) annotated 28 unique metabolites, predominantly lipids, nucleosides, and organic acids.

Shared metabolites between platforms are shown in the overlapping areas. Small overlaps revealed limited redundancy among techniques. These findings highlight the complementarity of the employed analytical platforms in covering a broader range of metabolite classes (see supplementary material S2. Table Metabolomics fermentation).

**Fig. 4:**
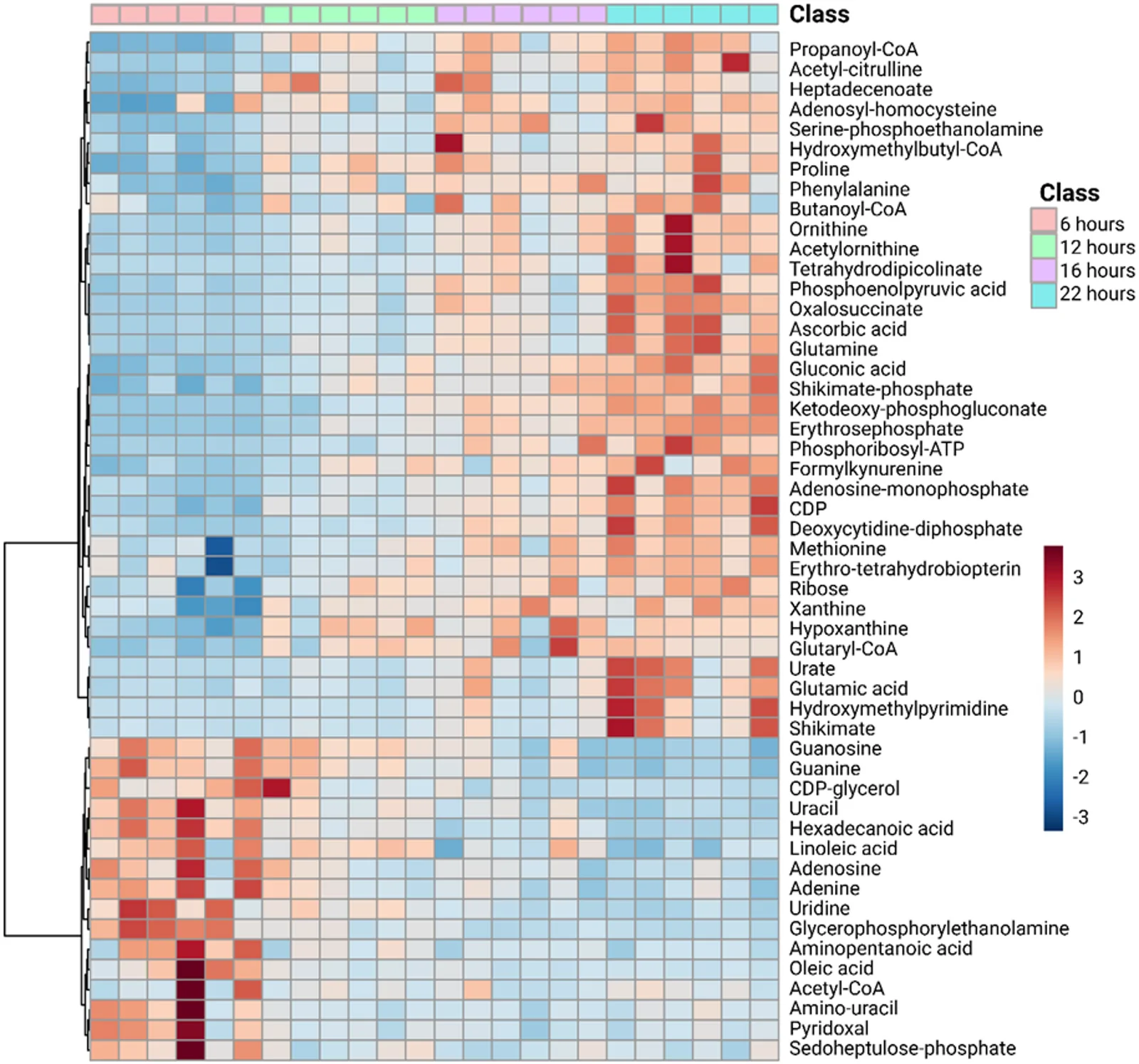
Heatmap of changes in the endometabolome of Burkholderia cepacia during the fermentation process using oleic acid as a carbon source. Expressions are normalized. Blue represents low expression and red represents high expression

To explore the dynamics of metabolite abundance during the fermentation process, a heatmap was generated using MetaboAnalyst 5.0 (Fig. 4). The identified metabolite database was first curated against the genomic information of the *B. cepacia* ATCC strain from KEGG and BioCyc databases, this analysis ensures the biological relevance of the analyzed metabolites.

The number of metabolites was reduced to 111 through curing, which was used to analyze metabolic pathways and interpret the metabolic changes occurring during the fermentation process. It is important to note that the omitted metabolites are primarily long-chain fatty acids that are not recorded in the aforementioned databases. Therefore, it was not possible to associate them with *B. cepacia* metabolic pathways for analysis. (See supplementary material S2. Table Metabolomics fermentation). The heatmap shows the relative abundance of metabolites with significantly abundance changes across the fermentation time points (6, 12, 16, and 22 h). Red colors indicate increased levels, while blue colors indicate decreased levels. The clustering analysis reveals two well-defined sets, each with distinct temporal patterns in metabolite levels.

The first set (at the top of the map) is characterized by metabolites that exhibit low abundance at the early stages of fermentation and progressively increase over time. This cluster displays a heterogeneous chemical composition, including a notable group of CoA derivatives such as propanoyl-CoA, butanoyl-CoA, and hydroxymethylbutyryl-CoA. Additionally, several amino acids such as proline, phenylalanine, glutamine, and ornithine along with carbohydrate intermediates like phosphoenolpyruvate, oxalosuccinate, and erythrose-phosphate are also present in this set. The elevated levels of amino acids and certain carbohydrates towards the end of the fermentation process could suggest metabolic changes involving pathways such as gluconeogenesis, pentose phosphate, and Entner-Doudoroff, which are directly related to energy production for cell growth and maintenance (Koller, 2020). These findings are in agreement with those described by Fukui et al. (2014), who observed that the use of octanoate as a carbon source in *R. eutropha H16* increased the concentration of acetyl-CoA, leading to the accumulation of carboxylic acids and the promotion of PEP and triose phosphate formation through oxaloacetate.

In contrast, the lower set exhibits the opposite trend, with metabolites present at higher levels during the early fermentation stages and decreasing over time. Notably, this group includes oleic acid, the supplied carbon source, which shows significant depletion, it suggests an intense endocytosis process, which exceeds the metabolic capacity to transform the substrate, in the initial moments of fermentation. Similarly, linoleic acid and hexadecanoic acid follow the same trend, reinforcing the idea of an early utilization of lipids metabolized by β-oxidation, one of the main pathways in *B. cepacia* for the production of energy and PHA when it has fatty acids as a carbon source (Escapa et al., 2012; Koller, 2020), and in agreement with what was described by Gao et al. (2011). This set also includes important metabolites such as nucleosides (adenosine, guanosine, uridine, adenine), pyridoxal, and CDP-glycerol. The elevated levels of nucleosides during early stages may reflect increased nucleotide turnover and biosynthetic activity for cell replication events, highlighting dynamic changes in nucleotide metabolism and cofactor availability as fermentation progresses.

Together, these results reveal a clear metabolic transition throughout the fermentation process. During the early stages, there is an intense degradation of fatty acids, such as oleic, linoleic, and hexadecanoic acids, which appear to serve as primary energy sources. As fermentation progresses, this metabolic pattern shifts toward the catabolism of amino acids and carbohydrates, as reflected by their increased abundance in later time points. This transition likely reflects an adaptive metabolic reprogramming in response to substrate availability and the energetic demands of the growing cells across the different phases of *B. cepacia* fermentation.

### Analysis of metabolic pathways during fermentation

It is important to consider that, in bacteria, PHA biosynthetic pathways are closely linked to core metabolic pathways, including glycolysis, TCA, β-oxidation, fatty acid degradation, amino acid catabolism, the pentose phosphate pathway, and the serine pathway (Koller, 2020; Tan et al., 2014). During fermentation, the dynamics of metabolic pathways in *B. cepacia* can be elucidated by analyzing the evolution of changes in time of the identified metabolites. The analysis of the metabolic pathways was performed by integrating an enrichment analysis with a pathway topology analysis. This enrichment analysis evaluated whether the identified metabolites were included within the metabolic pathways predicted theoretically from KEGG, using the genome of *Burkholderia mallei* ATCC 23,344. Pathway topology analysis assessed the relevance of each compound within the metabolic network, treating each metabolite as a node.

**Fig. 5:**
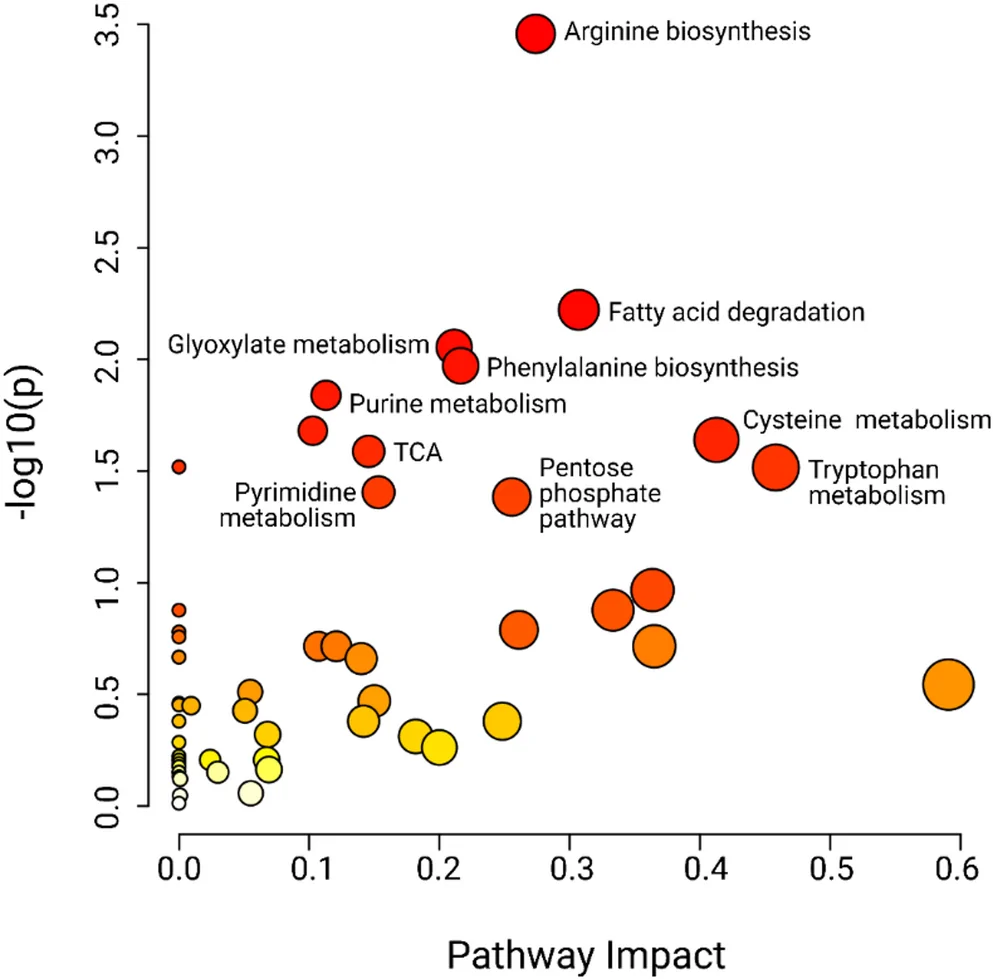
Analysis of metabolic pathways related to B. cepacia metabolism using oleic acid as carbon source. Made with MetaboAnalyst. The y-axis represents the enrichment of the pathway, and the x-axis represents the impact of the pathway. The size of the bubble is an indicator of the impact of the pathway and the hue indicates the enrichment

As illustrated in Fig. 5, these analyses enabled the identification of 58 distinct pathways. Of those pathways, 10 exhibited statistically significant enrichment, with an effect size greater than zero. Among these pathways, the fatty acid degradation pathway (β-oxidation), TCA, arginine biosynthesis, glyoxylate metabolism, phenylalanine metabolism, purine metabolism, cysteine metabolism, tryptophan metabolism, pyrimidine metabolism, and the pentose phosphate pathway are particularly noteworthy.

β-oxidation is the pathway through which fatty acids are metabolized under aerobic conditions (Sun et al., 2003; Thamarai et al., 2024). The subsequent analysis associated the following metabolites with this pathway: hexadecanoate, butanoyl-CoA, glutaryl-CoA, acetyl-CoA, dodecanoyl-CoA, decanoyl-CoA, hexanoyl-CoA, and octanoyl-CoA. The primary product of this metabolic pathway is acetyl-CoA, an intermediate that is shared by numerous core biosynthetic pathways in *B. cepacia* during PHA production (Koller, 2020). In PHA-producing microorganisms, such as *Cupriavidus necator*, *Chromatium vinosum*, and *Pseudomonas aeruginosa*, the metabolic flux from acetyl-CoA to PHA is found to be highly dependent on nutritional conditions (Steinbüche & Hein, 2001), this relationship is also observed in *B. cepacia*. The results observed in Fig. 4 describe an increase in β-oxidation intermediate metabolites in the exponential phase of the culture and an increase in the production of acetyl-CoA, suggesting that in this growth phase acetyl-CoA is channeled towards energy production and cell growth, and in turn, some β-oxidation intermediates such as (S)−3-hydroxyacyl-CoA are channeled towards PHA production, which explains a high biomass growth and polymer production since the exponential phase, confirming what was observed by Torres (2019). These results complement those described by (Tanadchangsaeng & Roytrakul, 2020) from an analysis of the *C. necator* proteome using glycerol as a carbon source, finding that the increase in enzymes related to gluconeogenesis reduces PHA production.

**Fig. 6:**
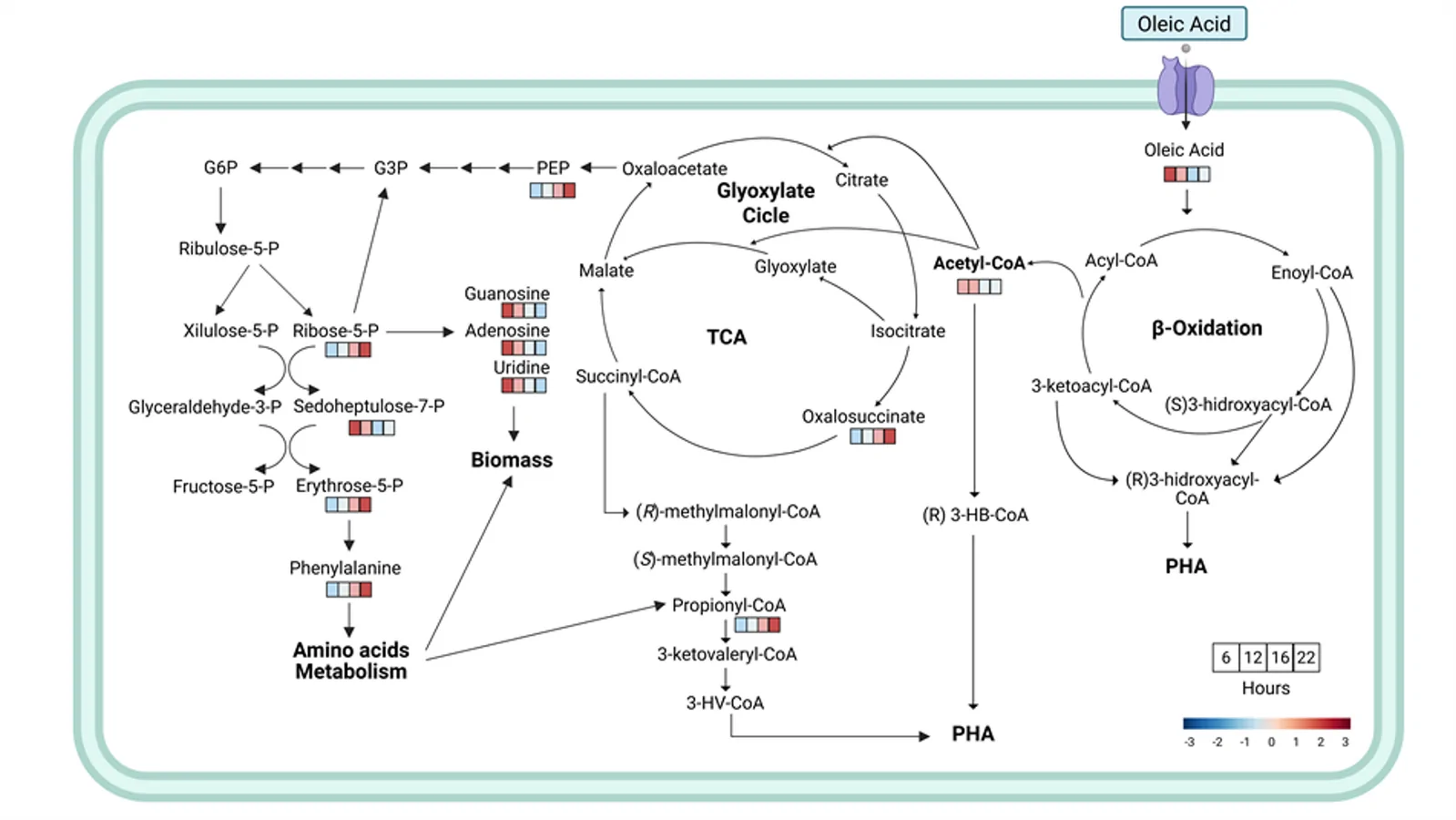
Metabolic pathways for PHA synthesis from oleic acid in *Burkholderia cepacia*. 3-HV-CoA (3-hydroxyvalerate-CoA), 3-HB-CoA (3-hydroxybutyrate-CoA), TCA (tricarboxylic acid), PEP (Phosphoenolpyruvate), G3P (Glyceraldehyde-3-phosphate), G6P (Glucose-6-phosphate)

Glucose anabolism is achieved through gluconeogenesis, a pathway activated by the glyoxylate metabolism, where PEP is generated from oxaloacetate by PEP carboxykinase (Muñoz-Elías & McKinney, 2006; Oh et al., 2002). This pathway activates the pentose phosphate pathway, an essential metabolic pathway for cell growth. Figure 6 shows higher abundances of some metabolites from gluconeogenesis and PP pathways such as PEP, erythrosephosphate (E4P), ribose (R5P) and phenylalanine in the stationary phase and the opposite behavior for the sedoheptulose-7-phosphate. These results are in agreement with those observed by Fukui et al. (2014), who studied the metabolism of *Ralstonia eutropha* (*Cupriavidus necator*) H16 during the PHA production phase with octanoate as a carbon source, that work suggest an activation of gluconeogenesis mediated by the activation of the glyoxylate cycle. The fluxomic analysis in *Burkholderia cepacia* with oleic acid (Torres-Ospina & Riascos, 2020) showed that gluconeogenesis is active at the exponential phase, allowing generation of E4P and R5P for phenylalanine and nucleotide acids, respectively, which are necessary for biomass growing. The metabolomic analysis, in the present work, shows lower abundance of E4P and R5P at growing phase (t = 6 and 12 h) and their accumulation when biomass production is reduced (t = 16 and 22 h), other interesting metabolites are adenosine, guanosine and uridine, which experience greater abundance in the growth phase (t = 6 and 12 h) and decrease between the end of the exponential phase and the stationary phase (t = 16 and 22 h). This behavior is contrary to that of metabolites such as R5P, which is a precursor for these nucleotides, suggesting that the regulatory mechanism could increase R5P abundance by blocking the generation of nucleotides. In future research, this could be investigated using multi-omics tools that facilitate the explanation of the interaction between these metabolites and their regulatory mechanisms.

Finally, the progressive increase in oxalosuccinate, suggesting an activation of TCA redirecting towards PHA production, between the exponential and stationary phases, allowing to maximize nutritional resources as an adaptation to the conditions of the culture medium. This ascending behavior is also observed in metabolites such as acetylornithine, glutamate and ornithine, suggesting the activation of metabolic pathways such as lysine degradation and glutamate metabolism, which contribute to the energetic metabolism of the microorganism when the nitrogen source decreases, which reaffirms what was described by Prieto et al. (2016), who describe that nitrogen limitations favor the production of PHA in *Pseudomonas putida* when the substrate is a fatty acid.

## Conclusions

A comprehensive analysis of the metabolome of *B. cepacia* is imperative to enhance our comprehension of the metabolic dynamics inherent to the fermentation process. The multiplatform strategy and the quality control procedures allowed to identify a wide set of metabolites with significant changes in their abundance during a batch fermentation for PHA production with *B. cepacia* using oleic acid as carbon source.

Metabolic pathway analysis enabled the identification of the most significant pathways *for B. cepacia* metabolism, emphasizing acetyl-CoA as the intermediate shared by the central biosynthetic pathways and PHA production. These findings confirmed the existence of a metabolic switch during the exponential phase, mediated by a complex regulation system that must be activated by changes in the concentrations of nutrients in the culture medium. This switch is the result of a metabolic network reorganization, thereby decreasing cell growth and redirecting the metabolism towards energy generation and polymer accumulation. This suggests that the microorganism undergoes this reorganization to optimize the use of the carbon source during cellular maintenance processes.

## Supplementary Information

Below is the link to the electronic supplementary material.

Supplementary Material 1

Supplementary Material 2

Supplementary Material 3

## Data Availability

All datasets generated during this study will be publicly available in the Metabolomics Workbench repository [https://www.metabolomicsworkbench.org](https:/www.metabolomicsworkbench.org). The corresponding accession numbers will be included in the final published version of the article.
