# Supplementary material for: Metabolomic characterization and temporal dynamics of Burkholderia cepacia for polyhydroxyalkanoates production from oleic acid
Source: Metabolomics. 2026 Jun 16;22(4):95. doi: 10.1007/s11306-026-02473-x (PMC13272603; doi:10.1007/s11306-026-02473-x)
Supplement: Supplementary file 2 — Supplementary Material 2 [file 11306_2026_2473_MOESM2_ESM.docx]

**Supplementary Material**

**
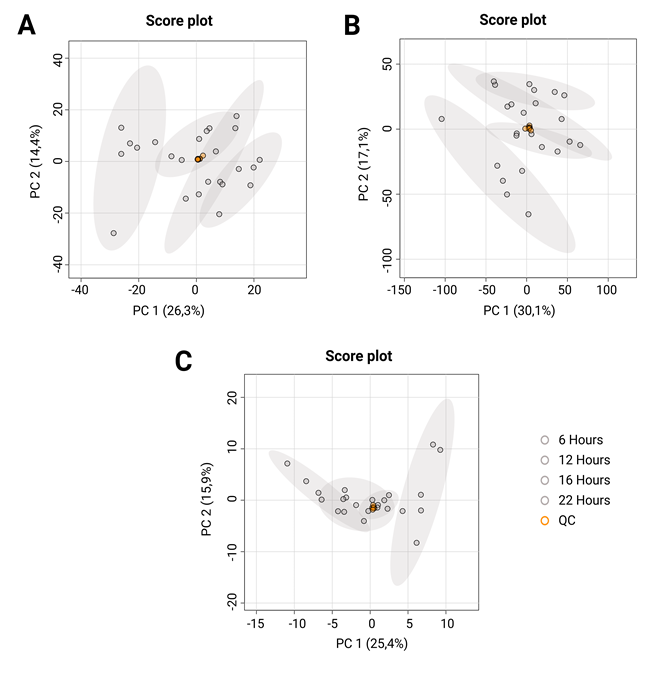
**

**Figure 1 Supplementary**: Unsupervised PCA models for the evaluation of the behavior of the QC. The orange ovals correspond to quality control (QC) samples, while the white ovals correspond to samples from different fermentation times.

The clustering of QC samples was observed for all analytical platforms, ensuring the quality of the acquired data and ensuring that the separation of groups is related to biological and non-analytical variations.
